# Effects of Positive Psychotherapy‐Based Education on Pain, Psychological Resilience, and Mental Well‐Being After Lumbar Canal Stenosis Surgery: A Randomized Controlled Trial

**DOI:** 10.1002/brb3.70873

**Published:** 2025-11-30

**Authors:** Ramazan Paşahan, Funda Kavak Budak, Serdar Saritaş, Mustafa Kavak, Fatma Melike Erkan, Sabır Akar

**Affiliations:** ^1^ Turgut Özal Medical Centre, Department of Neurosurgery İnönü University Malatya Turkey; ^2^ Department of Psychiatric Nursing İnönü University Malatya Turkey; ^3^ Department of Medical Biology Malatya Turgut Özal University Malatya Turkey; ^4^ Department of Surgical Nursing Erzurum Technical University Yakutiye Turkey; ^5^ Turgut Özal Medical Centre, Nursing İnönü University Malatya Turkey

**Keywords:** lumbar canal stenosis, mental well‐being, pain, positive psychotherapy, psychological resilience, surgery

## Abstract

**Aim:**

This study was conducted to investigate the effects of positive psychotherapy‐based education on pain, psychological resilience, and mental well‐being after lumbar canal stenosis surgery.

**Materials and Methods:**

A randomized controlled experimental study was conducted with 66 participants (33 control, 33 experimental) at the Brain and Neurosurgery Department of Medical Center X between March 2025 and July 2025. The control group received no intervention, while the experimental group received 8 weeks of psychoeducation based on positive psychotherapy. Pretests were administered to the control and experimental groups in March 2025. The training of the experimental group was administered between April 2025 and May 2025. Data collection tools included the Descriptive Characteristics Form, Pain Scale, Brief Resilience Scale, and Mental Well‐Being Scale. It was determined that the data showed normal distribution, and parametric tests were used in the analyses. Chi‐square and Fisher's exact tests were used to compare the descriptive characteristics of the patients in the experimental and control groups. A *t*‐test was applied to the dependent and independent groups to determine the effect of the training.

**Findings:**

In the study, no statistically significant difference was found in the comparison of the pretest pain, psychological resilience, and mental well‐being scales of the control and experimental groups (*p* = 0.259, *p* = 0.177, *p* = 0.428). In the study, a statistically significant difference was found in the comparison of the posttest pain, psychological resilience, and mental well‐being scales of the control and experimental groups (*p* = 0.001). In the study, a statistically significant difference was found in the pretest–posttest pain, psychological resilience and mental well‐being scale total score averages of the experimental group (*p* = 0.001).

**Conclusion and Recommendations:**

Positive psychotherapy‐based education was effective in reducing pain and increasing psychological resilience, and mental well‐being in patients after lumbar spinal stenosis surgery. Based on these findings, positive psychotherapy‐based education can be recommended as an adjunct to pharmacological treatment for not only physical but also psychological recovery.

## Graphical abstract

This study was conducted to investigate the effects of positive psychotherapy‐based education on pain, psychological resilience, and mental well‐being after lumbar canal stenosis surgery. The control group received no intervention, while the experimental group received 8 weeks of psychoeducation based on positive psychotherapy. Pretests were administered to the control and experimental groups in March 2025. The training of the experimental group was administered between April 2025 and May 2025. Data collection tools included the Descriptive Characteristics Form, Pain Scale, Brief Resilience Scale, and Mental Well‐Being Scale. Positive psychotherapy‐based education was effective in reducing pain and increasing psychological resilience and mental well‐being in patients after lumbar spinal stenosis surgery.

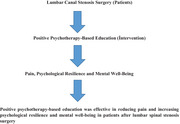

## 1 Introduction

Lumbar canal stenosis (LCS), defined as the compression of neural structures, is defined as the narrowing of the spinal canal caused by the compression of soft and bony tissues. A total of 130 million of the world's population suffer from LCS. In the United States of America (USA), approximately 600,000 cases require surgical treatment each year. According to the Turkish Health Survey data, back‐related disorders (back pain, lumbar disc herniation, and other back defects) experienced by individuals aged 15 and over in the last year are the leading health problems, with 24.6% (Katz et al. 2022; Türkiye İstatistik Kurumu 2022; Darlow et al. 2022; Karaeminoğulları and Aydınlı 2004). There are conservative and surgical treatments for LCS to increase the patient's functionality and reduce pain (Katz et al. 2022). Every year, 230 million people in the world undergo surgical intervention (Ay and Evcik 2007; Müjde et al. 2015; Zahn et al. 2017). Approximately 80% of patients who undergo intervention report experiencing pain after surgery (Apfelbaum et al. 2003).

In spine surgery, the size of the skin incision, the use of surgical implants, and the prolonged duration of surgery cause pain (Kim et al. 2015). In their studies, Iorio et al., Fritz et al., and Kalichman et al. found that pain continued in the long term after LCS surgery (Kim et al. 2015; Coronado et al. 2015). After the surgical procedure, patients are affected not only by physical but also by psychological factors. Studies show that the psychological well‐being of patients is negatively affected after LCS (Jackson et al. 2020; McGregor et al. 2014; Wilhelm et al. 2017). The improvement in psychological well‐being after LCS is closely related to the concept of psychological resilience.

Psychological resilience enables patients to cope with the stress and emotional challenges they encounter during the recovery process. Strong psychological resilience can help patients approach the rehabilitation process in a more positive way. Studies have shown that patients with high psychological resilience recover more quickly physically and mentally after surgery (Miller et al. 2024; Petrucci et al. 2025; Abbott et al. 2010).

Various nonmedical treatments are used to improve both physical and mental health after surgery. Postsurgical pain not only has physical effects but also has psychological, emotional, and behavioral effects. Therefore, it is important to implement nonmedical treatments after surgery. Some of the non‐pharmacological methods used after LCS surgery are distraction, virtual reality, music therapy, psychotherapies, and breathing exercises. Among psychotherapy methods, cognitive behavioral therapies and positive psychotherapy are frequently preferred (Koraş and Karabulut 2018; Ferhat 2016; John et al. 2022; Gündoğdu Altın 2022). Positive psychotherapy can be effective on both physical pain and mental well‐being and psychological resilience by instilling hope for the future, using positive thinking and coping strategies to cope with difficulties.

Positive psychotherapy‐based education is one of the most widely used therapy methods today. The aim of this education is to diversify the patient's coping methods (Boessmann and Remmers 2022). The word “positive” in positive psychotherapy doesn't just mean positive; it also means seeing things as they are. The primary focus of this therapy is to find the positive, reinterpret symptoms from a positive perspective, and help the patient achieve balance in their life (Peseschkian 2023). One study found that individuals who participated in this training experienced a decrease in pain and an increase in psychological resilience (Maffei 2020). Another study observed improvements in mental and physical health and quality of life, as well as reductions in pain and stress levels (Nasiri Takami et al. 2020). Positive psychotherapy, with its holistic (bio‐psycho‐social) and therapeutic approach, evaluates patients holistically. Concepts such as looking to the future with hope, forgiving oneself and those around them, feeling grateful, and learning to cope with challenges, which constitute the core of positive psychotherapy, are particularly important for patients following lumbar spinal stenosis surgery. It is believed that positive psychotherapy will benefit these patients' physical and mental well‐being, as they may experience immobility, pain, and psychological distress after surgery.

A review of the literature reveals that positive psychotherapy‐based training has been applied to groups with various conditions, and its effects on the parameters examined in our study have been reported to be beneficial. However, no studies have been found examining the effects of positive psychotherapy‐based training on pain, psychological resilience, and mental well‐being after surgery in patients undergoing lumbar spinal stenosis surgery, which we selected. In this regard, our research is expected to contribute to the literature.

This study was conducted to examine the effects of positive psychotherapy‐based training on pain, psychological resilience, and mental well‐being after LCS surgery.

### 1.1 The Hypotheses of the Study

*H_1a_: Positive psychotherapy‐based education given to the experimental group after LCS surgery reduces the pain level of the patients compared to the control group*.

*H_1b_: Positive psychotherapy‐based education given to the experimental group after LCS surgery increases the psychological resilience level of the patients compared to the control group*.

*H_1c_: Positive psychotherapy‐based education given to the experimental group after LCS surgery increases the mental well‐being level of the patients compared to the control group*.

## 2 Method

### 2.1 Research Type

This study was conducted as a randomized controlled experimental study.

### 2.2 Research Location and Time

The study was conducted between March 2025 and July 2025 at the Brain and Neurosurgery Department of Medical Center X.

### 2.3 The Universe and Sample of the Study

The universe of the study consisted of patients who underwent LCS surgery in the neurosurgery department. In this study, a priori power analysis was performed for each of the pain, psychological resilience, and mental well‐being levels using the G Power 3.1.9.4 program in calculating the sample size. The studies conducted were examined (Firdous et al. 2019), and the expected confidence intervals for the “Pain Score Evaluation” were determined, and the confidence interval was calculated as *α* = 0.05, the power of the test (1−β) was 0.95, the effect size was *d* = 0.8232193, and 60 participants were included in the experimental group, and 30 in the control group.

Studies conducted for the Psychological Resilience Scale were examined (Petrucci et al. 2025), and the expected confidence intervals of the “Psychological Resilience Scale” were determined. The confidence intervals were determined as *α* = 0.05, the power of the test (1−β) was 0.95, the effect size was *d* = 1.022014, and 50 patients were determined as 25 in the experimental group and 25 in the control group. In addition, studies conducted for the mental well‐being scale were examined (Asher et al. 2015), and the expected confidence intervals of the “Mental Well‐being Scale” were determined, and the confidence intervals were calculated as *α* = 0.05, the power of the test (1−β) was 0.95, the effect size was *d* = 0.8914242, and 56 patients were included in the experimental group, and 28 in the control group. As a result of the power analysis, the sample size of the study was determined to be 60 patients in total, 30 in the experimental group and 30 in the control group, since the sample size was found to be higher in the Pain Score Evaluation. Considering the possibility of patients not continuing the study for various reasons during the implementation phase of the study, and in order to preserve the statistical power of the study, it was planned to conduct the study with approximately 20% more than the sample, 72 participants (36 experimental, 36 control group). The study was finally completed with 66 participants (33 experimental, 33 control).

Inclusion Criteria for the Study: being between the ages of 18 and 65, having received general anesthesia, being on the first zeroth day after surgery, receiving inpatient treatment, patients who underwent stabilization (Levels 2–4), and patients who underwent stenosis surgery without stabilization.

Exclusion Criteria for the Study: recurrent surgery, having had previous back surgery, degenerative scoliosis patients, having a rheumatological disease, developing any complications such as nausea, severe bleeding, vomiting, and so forth in the early period after surgery, having any mental illness, and having attended a training related to positive psychotherapy at least 6 months ago.

Exclusion Criteria from the Study: having had unsuccessful back surgery, wanting to leave the study during the study period, or not being able to complete the training sessions for different reasons.

## 3 Data Collection Tools

### 3.1 Descriptive Characteristics Form

This form consists of seven questions, including parameters such as age, education level, gender, marital status, employment status, mobility status, and previous treatment/surgery status.

#### 3.1.1 Numerical Rating Scale

This scale starts with the absence of pain at “0” and ends the unbearable pain at “10–100”. In this study, the Numerical Rating Scale (NRS) numbered 0–10, was used.

#### 3.1.2 Connor–Davidson Psychological Resilience Scale Short Form

The adaptation of the scale developed by Connor and Davidson (2003) to Turkish was conducted by Kaya and Odacı (2021). It was stated that the internal consistency coefficient of the scale consisting of 10 items was 0.81. A high score from the scale indicates high psychological resilience (Kaya and Odacı 2021). In this study, Cronbach's alpha coefficient of the scale was determined to be 0.89.

#### 3.1.3 Warwick–Edinburgh Mental Well‐Being Scale

The Turkish validity and reliability study of the scale developed by Tennant et al. (2007) was conducted by Keldal (2015) (Asher et al. 2015; Connor and Davidson 2003). The scale is a 5‐point Likert‐type scale, and the minimum score is 14, and the maximum score is 70. High scores obtained from the scale indicate high mental well‐being (Tennant et al. 2007; Keldal 2015). Cronbach's alpha coefficient of the scale was determined to be 0.86. In this study, Cronbach's alpha coefficient of the scale was determined to be 0.90.

#### 3.1.4 Randomization

In this study, the block randomization method was used to ensure a balanced number of participants in the experimental and control groups. In the block randomization method, six different block combinations were created, consisting of blocks of four containing the letters A and B. The numbers from 1 to 6 were ranked using the simple random assignment method (https://www.randomizer.org/) until the number obtained by dividing the numbers by the number of options in the combination within the sample number (80/4 = 20). Blocks of four were placed according to the order created. The experimental or control group, represented by the letters A and B, was determined by the lottery method. The patients were numbered according to the file number in the service. The experimental and control groups were assigned according to the blocks created as a result of the order.

#### 3.1.5 Data Collection

Data were collected by the researchers through face‐to‐face interviews at the neurosurgery department of Medical Center X, with an initial interview followed by planned training sessions in the meeting room. The administration of the data collection tools took approximately 20 min.

#### 3.1.6 Research Process

Patients participating in the study were referred to the researcher by neurosurgery physicians. Participants were initially informed about the purpose and scope of the study. Written and verbal consent was obtained from participants who agreed to participate in the study. Participants who met the inclusion criteria were randomly assigned to the experimental and control groups. Pretests (Descriptive Characteristics Form, Pain Scale, Psychological Resilience Scale, and Mental Well‐being Scale) were applied to the participants in the experimental and control groups before starting the training sessions. The 8‐week positive psychotherapy‐based training was applied to the patients in the experimental group in the meeting room of the service once a week in the form of group training, each training session lasting 45 min. The training sessions consisted of an average of 5–6 people.

Posttests (Pain Scale, Psychological Resilience Scale, and Mental Well‐being Scale) were administered to the patients in the experimental group immediately after the training sessions were completed. Posttests (Pain Scale, Psychological Resilience Scale, and Mental Well‐being Scale) were applied to the patients in the control group at the eighth week without any additional intervention other than routine treatment. The patients in the control group were given a positive psychotherapy‐based education booklet after the posttest.

#### 3.1.7 Positive Psychotherapy‐Based Training

Positive psychotherapy training is based on Barbara Fredrickson's theory of expanding and creating positive emotions. According to this theory, positivity in emotions is effective in positive thoughts. For example, experiencing feelings of joy, hope, and peace can positively influence individuals' thoughts and behaviors. Positive psychotherapy plays a significant role in positively influencing the emotions and thoughts of patients undergoing lumbar spinal stenosis surgery, reducing physical pain and promoting psychological recovery.

The positive psychoeducation program, developed by the researchers based on a literature review, consisted of one session per week over 8 weeks. Each session lasted approximately 45 min in group instruction. The researcher who provided the training has a doctorate in psychiatry and has received training in positive psychotherapy and cognitive behavioral therapy. He is an expert in his field. To ensure a standardized approach to each session, the researchers developed an implementation guide. Each session included storytelling, live role‐playing, group discussions, and homework assignments. At the beginning of each session, the assigned homework assignments were reviewed before moving on to the next session.

The researcher conducting the training conducted verbal psychological assessments of the patients before and after each session. Patients who were feeling unwell were given breathing exercises and emotional focus. Patients who were not relieved by these practices were referred to a specialist psychiatrist for psychological support.

The sessions consist of the following topics:

1. *Introduction to Positive Psychotherapy and Basic Concepts*: In this session, patients were introduced. Detailed information about positive psychotherapy training was provided. They were also briefly briefed on their illnesses.
2. *Perception, Balance Model, and Core Competencies*: This session helped patients become aware of their emotions. They discussed their perspectives on their lives, particularly how they perceive them, and became aware of their negative perceptions.
3. *Recognizing Individual Strengths*: This session aims to foster self‐awareness. It aims to help individuals recognize their strengths and see that they can achieve much in life.
4. *Ways of Coping with Challenges*: In this session, patients were taught methods for coping with stress and anxiety. Individuals experiencing mental health difficulties were taught new coping methods in addition to their old ones.
5. *Meaning and Goal Setting*: In this session, patients were taught to focus on other important things in their lives, apart from their illness. They were encouraged to become aware of what was meaningful in their lives outside of their illness.
6. *Positive Approaches to Relationships*: This session emphasized the importance of therapeutic communication and the importance of interpersonal relationships in our lives. It explained that connecting with people can provide psychological relief. Effective communication techniques were taught through examples.
7. *Developing Forgiveness, Gratitude, and Hope*: In this session, patients were taught to look to the future with hope and not to regret past events. Exercises were used to foster daily gratitude, along with stories designed to foster hope.
8. *General Assessment and Maintenance of Gains*: In this session, a general evaluation was made, and the effectiveness of the training was discussed.

The training applied to the experimental group was carried out in the form of interactive training supported by a training booklet (Table 1).

**TABLE 1:** Positive psychotherapy‐based education implementation plan.

1.Session: Introduction to positive psychotherapy and basic concepts (observation/distance period)	2.Session: introduction to positive psychotherapy and basic concepts (inventory period)
Meeting patients Introducing the content of the training Emphasizing the importance of regular and timely attendance at training Determining training days and times Sharing patients' expectations	What is lumbar stenosis? What are the treatment methods? How does the disease progress? What should be done during and after the surgical procedure?
**3. Session: perception, balance model, and core competencies**	**4. Session: recognizing individual strengths**
Participants' awareness of the functions of their symptoms and their effects on daily life within the framework of the Balance Model, Participants' expression of their reactions to their symptoms and the desired alternative response to each within the framework of the Balance Mode	Participants' awareness of their patterns and how early childhood experiences affect their current symptoms and lives.
**5. Session: ways of coping with challenges**	**6. Session: positive approaches to relationships**
To have participants realize the concepts of family that were very useful in early childhood but are now dysfunctional and do not meet their emotional needs, To have participants realize the content of their basic conflicts, To discuss what can be done to make dysfunctional relationship cycles functional and what may happen as a result.	Providing information to participants about positive interpretation in the PPT, Encouraging participants to interpret pain from alternative perspectives.
**7. Session: meaning and goal setting/developing forgiveness, gratitude, and hope**	**8. Session: general assessment and maintenance of gains**
Introduce users to basic emotions, Teach participants to recognize and name their emotions, Improve participants’ ability to answer the “How Am I Feeling” question, Encourage participants to reflect on their feelings about their symptoms, Help participants discover the characteristics of recurring dysfunctional thoughts, Help participants discover dysfunctional thoughts and learn to transform them into positive thoughts.	Helping participants become aware of their stress sources and stress reactions, Providing participants with information about stress management and teaching them how to use it in their lives. Evaluating the program. Obtaining feedback.

#### 3.1.8 Statistical Analysis of Data

Statistical analyses of the study were performed using the SPSS 22 package program. Percentage, frequency, arithmetic mean, and standard deviation were used to determine the descriptive characteristics of the patients in the analysis. The conformity of the data to normal distribution was examined with skewness and kurtosis coefficients (between −1.5 and +1.5). It was determined that the data showed normal distribution, and parametric tests were used in the analyses. Chi‐square tests were used to compare the descriptive characteristics of the patients in the experimental and control groups. A *t*‐test was applied to the dependent and independent groups to determine the effect of the training. Cronbach's alpha coefficient was calculated to determine the reliability levels of the scales used in the study. The significance level in the analyses was determined as *p* < 0.05.

#### 3.1.9 Ethical Principles of the Research

Ethics committee approval was obtained from the University Ethics Committee of Y in order to conduct the research (IRB NO: 2025/7531).

In addition, written permission was obtained from the institution where the research was conducted (2025‐572110). Patients included in the study were informed about the research and signed the Informed Consent Form.

### 3.2 Findings

When the control and experimental groups were compared in terms of descriptive characteristics, it was determined that there was no statistically significant difference between them (*p* > 0.05, Table 2). It can be said that the control and experimental groups were homogeneous in terms of descriptive characteristics.

**TABLE 2:** Distribution and comparison of descriptive characteristics of the patients in the control and experimental groups.

Descriptive characteristics	Control (*n* = 33)		Experimental (*n* = 33)		Chi‐Square, *φ* and *p* value
	*n*	%	*n*	%	
Age					
20–30	4	12.12	5	15.15	*χ* ^2^(1, 33:):1.133 *p* = 0.583 *φ* = 0.19
31–41	9	27.27	10	30.30	
42 yaş ve üzeri	20	60.60	18	54.54	
Gender					*χ* ^2^(1, 33:):1.828 *p* = 0.369 *φ*:0.24
Female	22	66.66	21	63.63	
	11	33.33	12	36.36	
Male					
Educational level					
Primary school Secondary school and above	13	39.39	15	45.45	*χ* ^2^(1, 33:):2.273
	20	60.60	18	54.54	*p* = 0.074 *φ*:0.26
Medeni durum					
Evli	25	75.75	24	72.72	*χ* ^2^(1, 33:):2.267
	8	24.24	9	27.27	*p* = 0.114 *φ*:0.26
Bekar					
Çalışma durumu					
Çalışıyor	7	21.21	8	24.24	*χ* ^2^(1, 33:):2.322
	26	78.78	25	75.75	*p* = 0.606 *φ* = 0.27
Çalışmıyor					
Hareket durumu					
Serbest	3	9.09	2	6.06	*χ* ^2^(1, 22:):2.773 *p* = 0.428 *Φ* = 0.36
Kısıtlı	9	27.27	10	30.30	
Kısmen kısıtlı	21	63.63	21	63.63	
Daha önce tedavi aldı mı					
Evet	23	69.69	22	66.66	*χ* ^2^(1, 33:) = 1.773 *p* = 0.228 *Φ* = 0.23
Hayır	10	30.30	11	33.33	

*Note: “p* < 0.05 is significant”, *α* = 0.05.

* Chi‐square tests.

In the study, no statistically significant difference was found in the comparison of the pretest pain, psychological resilience, and mental well‐being scales of the control and experimental groups (*p* = 0.259, *p* = 0.177, *p* = 0.428, Tables 3, 4, 5). In the study, a statistically significant difference was found in the comparison of the posttest pain, psychological resilience, and mental well‐being scales of the control and experimental groups (*p* = 0.001, Tables 3, 4, 5). It was determined that there was a statistically significant difference in the posttest pain, psychological resilience, and mental well‐being level of the experimental group compared to the control group (*p* = 0.001, Tables 3–6).

**TABLE 3:** Comparison of pretest–posttest total mean scores of experimental and control groups on the Pain Scale.

		Pretest	Posttest	Test, Cohen's *d* and *p* value^a^
**Total mean score of Pain Scale**	Control group (*n* = 33)	7.50 ± 0.97	3.70 ± 0.67	*t*(33) = 2.334 ***p*** = **0.001** Cohen's *d* = 0.82
	Experimental group (*n* = 33)	7.50 ± 0.84	1.20 ± 0.68	*t*(33) = 3.451 ***p*** = **0.001** Cohen's *d* = 0.94
	Test value and *p* value^b^	*t* = 2.334 *p* = 0.259	*t* = 1.928 ***p*** = **0.001**	

*Note*:  *α* = 0.05, *p* < 0.05 is significant.

^a^ Independent samples *t*‐test.

^b^ Dependent samples *t*‐test.

**TABLE 4:** Comparison of pretest–posttest total mean scores of experimental and control groups on the Psychological Resilience Scale.

		Pretest	Posttest	Test, Cohen's *d* and *p* value^a^
Total mean score of Psychological Resilience Scale	Control group	9.60 ± 2.16	10.40 ± 3.69	*t*(33) = 1.185 *p* = 0.064 **Cohen's *d*** = **0.26**
	Experimental group	9.20 ± 2.87	34.50 ± 7.83	*t*(33) = 3.603 ***p*** = **0.001** Cohen's *d* = 3.26
	Test value and *p* value^b^	*t* = 1.154 *p* = 0.177	*t* = 1.499 ***p*** = **0.001**	

*Note*: *α* = 0.05, *p* < 0.05 is significant.

^a^ Independent samples *t*‐test.

^b^ Dependent samples *t*‐test.

**TABLE 5:** Comparison of pretest–posttest total mean scores of experimental and control groups on the Mental Well‐Being Scale.

		Pretest	Posttest	Test, Cohen's *d* and *p* value^a^
Total mean score of Mental Well‐Being Scale	Control group	22.40 ± 2.35	23.60 ± 2.57	*t*(33) = 1.366 *p* = 0.074 Cohen's *d*:0.48
	Experimental group	22.30 ± 2.11	56.90 ± 10.12	*t*(33) = 3.344 ***p*:0.001** Cohen's *d* = 3.65
	Test value and *p* value^b^	*t* = 1.928 *p* = 0.428	*t* = 1.334 ***p*** = **0.001**	

*Note: α* = 0.05, *p* < 0.05 is significant.

^a^ Independent samples *t*‐test.

^b^ Dependent samples *t*‐test.

**TABLE 6:** Summary Findings.

	Control group*		Experimental group**		Test, Cohen's *d* and *p* value**
	Pretest	Posttest	Pretest	Posttest	
Total mean score of Mental Well‐Being Scale	22.40 ± 2.35	23.60 ± 2.57	22.30 ± 2.11	56.90 ± 10.12	*t(33) = 1.366 *p* = 0.074 Cohen's *d* = 0.48 **t(33) = 3.344 ***p*** = **0.001** Cohen's *d* = 3.65
Total mean score of Pain Scale	7.50 ± 0.97	3.70 ± 0.67	7.50 ± 0.84	1.20 ± 0.68	*t(33) = 2.334 ***p*** = **0.001** Cohen's *d* = 0.82 **t(33) = 3.451 ***p*** = **0.001** Cohen's *d* = 0.94
Total mean score of Psychological Resilience Scale	9.60 ± 2.16	10.40 ± 3.69	9.20 ± 2.87	34.50 ± 7.83	*t(33) = 1.185 *p* = 0.064 **Cohen's *d*** = **0.26** **t(33) = 3.603 ***p*** = **0.001** Cohen's *d* = 3.26

*Note: α* = 0.05, “*p* < 0.05” is significant.

^a^ Independent samples *t*‐test.

## 4 Discussion

The findings of the study conducted to examine the effects of positive psychotherapy‐based education on pain, psychological resilience, and spiritual well‐being after LCS surgery were discussed in line with the literature.

In the study, it was determined that the pain levels of the individuals in the control and experimental groups before the training were above average. Studies show that pain continues to be a major problem after surgical intervention (Zahn et al. 2017; Apfelbaum et al. 2003; Kim et al. 2015). Studies have indicated that pain in patients after spinal surgery is above average (Zahn et al. 2017; Apfelbaum et al. 2003; Kim et al. 2015; Coronado et al. 2015). The fact that a very short time has passed since the surgical procedure and the patient's concerns may be considered as the reason for this result. The research result is parallel to the literature.

In the study, it was determined that there was a statistically significant decrease in the pain levels of the individuals in the control and experimental groups after the training, and the decrease was greater in the experimental group than in the control group. Research finding supports the hypothesis *H_1a_: Positive psychotherapy‐based training given to the experimental group after LCS surgery reduces the pain level of the patients compared to the control group*. It is thought that the decrease in pain level in the control group may be related to reasons such as the passage of time since the surgical procedure, the decrease in the patient's movement limitation, and the decrease in their anxiety. In studies conducted on patients with chronic low back pain, psychoeducation‐based interventions have been shown to reduce pain severity and anxiety symptoms (Karaokur 2019). In the current study, it was observed that including not only physical intervention but also psychological support programs could be beneficial in terms of a holistic treatment approach. In the literature, it is frequently emphasized that chronic pain after surgery is not only related to physical but also emotional and cognitive processes (Zahn et al. 2017; Apfelbaum et al. 2003; Kim et al. 2015; Coronado et al. 2015; Jackson et al. 2020). Positive psychotherapy‐based education aims to improve stress coping skills by focusing on the individual's positive aspects and their capacity to find meaning in life. In this context, research results show that education can modulate postsurgical pain perception and support the recovery process of patients.

The study found that individuals in the control and experimental groups had low levels of psychological resilience before the training. Graffigna and Barello (2018) emphasize that critical health conditions profoundly affect patients' emotional well‐being and psychological resilience. Psychological resilience refers to the individual's capacity to maintain psychological balance and adapt to stressful life events. Major surgical procedures, such as LCS surgery, can pose both a physical and psychological burden for individuals (Petrucci et al. 2025; Asher et al. 2015). Therefore, it can be said that patients have low levels of psychological resilience.

While no statistically significant difference was found in the psychological resilience levels of individuals in the control group after the training, the difference found in the experimental group was statistically significant. The psychological resilience level of individuals in the experimental group increased after the training. Research finding *H_1b_: Positive psychotherapy‐based training given to the experimental group after LCS surgery increases the psychological resilience level of the patients compared to the control group*. In this study, positive psychotherapy training and information about the disease process, along with informing the patient about what to expect, strengthened the individual's sense of control. Education was provided to address the patient's uncertainties regarding the surgical procedure and its aftermath. In addition, the patient was provided with the development of active coping strategies to be stronger against negativities and the feeling of not being alone by communicating with the healthcare team. In this context, positive psychotherapy training applied in the postsurgical period contributed to the development of positive feelings and thoughts of individuals, to the reconstruction of their sense of meaning and purpose, and supported psychological resilience.

In the study, it was determined that the mental well‐being levels of the individuals in the control and experimental groups before the training were below average. Mental well‐being is important for the continuation of life, both individually and environmentally and socially. Individuals with high levels of mental well‐being have better psychological and physical health and can establish good relationships with other people. The intense anxiety experienced by patients before the surgical procedure and the pain, uncertainty, fear of getting sick again, and physical changes experienced after the surgical procedure can negatively affect mental health. In this context, the low mental well‐being levels of the patients are an expected result.

While no statistically significant difference was found in the psychological well‐being levels of the individuals in the control group after the training, the difference found in the experimental group was statistically significant. The psychological well‐being levels of the individuals in the experimental group increased after the training. Research finding H1c: Positive psychotherapy‐based training given to the experimental group after LCS surgery increases the mental well‐being level of the patients compared to the control group. Positive psychotherapy is based on the individual discovering their strengths, values, and meaning in life rather than focusing solely on symptoms. The training program implemented in this study is thought to strengthen individuals’ self‐perception, increase life satisfaction, and support positive posttraumatic adaptation skills (Seligman et al. 2005). When the psychological difficulties experienced after medical interventions that limit physical functionality, such as lumbar stenosis surgery, are taken into account, the importance of such psychosocial support programs becomes even more apparent. It has been observed that emotional burdens such as helplessness, social isolation, and anxiety about the future, which are commonly observed in individuals undergoing surgery, are reduced with positive psychotherapy training (Seligman et al. 2005; Rashid and Seligman 2018). This situation reveals that the way individuals make sense of life events plays a decisive role in psychological well‐being.

This study found that positive psychotherapy‐based training provided to patients after lumbar spinal stenosis surgery reduced pain and positively increased psychological resilience and mental well‐being. The content of the positive psychotherapy‐based training plays a significant role in this outcome. Concepts such as self‐awareness and adding meaning to their lives by changing their perspectives on life and themselves, which are included in the training content, play a particularly important role in increasing psychological resilience. Furthermore, concepts such as looking to the future with hope, developing new coping strategies to cope with challenges, using effective communication techniques in interpersonal relationships, and fostering positive thinking by becoming aware of one's own feelings and thoughts positively impact patients' mental well‐being. Because positive psychotherapy treats individuals holistically, these aspects also positively impact physical health.

### 4.1 Conclusion and Recommendations

In this study, considering the total scale mean scores of the patients before the positive psychotherapy‐based training, it was observed that their pain levels were high, while their psychological resilience and mental well‐being levels were low. This suggests that patients experience both physical and psychological distress after lumbar spinal stenosis surgery. A study conducted to evaluate the effects of positive psychotherapy‐based training on pain, psychological resilience, and mental well‐being in individuals undergoing LCS surgery found that individuals who received positive psychotherapy training experienced a significant reduction in pain levels, a significant increase in psychological resilience scores, and positive improvements in mental well‐being. These findings reveal that not only physical rehabilitation but also psychological support is important in the postsurgical process. Positive psychotherapy‐based approaches contribute to the healing process and increase the quality of life by improving individuals' ability to cope with difficulties. In line with these results, it can be said that positive psychotherapy‐based interventions can play a supportive and complementary role in postsurgical care processes. Such approaches should be part of a holistic health approach that targets not only the physical but also the psychological well‐being of individuals.

### 4.2 Limitations of the Study

The study was conducted in a specific center and with a limited number of participants. This may limit the generalizability of the results. It may be recommended to conduct larger and more multicenter studies. The effects of education were evaluated in the short term. It may be recommended to evaluate the effects of education on pain, psychological resilience and spiritual well‐being in the long term. Another limitation is that the study did not use a blinding method and did not conduct follow‐up tests.

## Author Contributions

**Ramazan Paşahan**: investigation, validation, methodology. **Funda Kavak Budak**: methodology, validation, writing – review and editing. **Serdar Saritaş**: visualization, formal analysis, writing – original draft. **Mustafa Kavak**: funding acquisition, formal analysis, software. **Fatma Melike Erkan**: data curation, resources, project administration, formal analysis. **Sabır Akar**: supervision, conceptualization.

## Conflicts of Interest

The authors declared no conflicts of interest.

## Data Availability

The data used to support the findings of this study are available from the corresponding author on request.

## References

[brb370873-bib-0001] Abbott, A. D. , R. Tyni‐Lenné , and R. Hedlund . 2010. “The Influence of Psychological Factors on Pre‐Operative Levels of Pain Intensity, Disability and Health‐Related Quality of Life in Lumbar Spinal Fusion Surgery Patients.” Physiotherapy 96, no. 3: 213–221. 10.1016/j.physio.2009.11.016. 20674653

[brb370873-bib-0002] Apfelbaum, J. L. , C. Chen , S. S. Mehta , and T. J. Gan . 2003. “Postoperative Pain Experience: Results From a National Survey Suggest Postoperative Pain Continues to be Undermanaged.” Anesthesia & Analgesia 97, no. 2: 534–540. 10.1213/01.ANE.0000068822.10113.9E. 12873949

[brb370873-bib-0003] Asher, R. , A. E. Mason , J. Weiner , and R. G. Fessler . 2015. “The Relationship Between Preoperative General Mental Health and Postoperative Quality of Life in Minimally Invasive Lumbar Spine Surgery.” Neurosurgery 76, no. 6: 672–679. 10.1227/NEU.0000000000000682. 25714515

[brb370873-bib-0004] Ay, S. , and D. Evcik . 2007. “Spinal Stenoz Ve Fizik Tedavi Yaklaşımları.” Journal of Turkish Spinal Surgery 18: 57–66.

[brb370873-bib-0005] Boessmann, U. , and A. Remmers . 2022. Pozitif Psikodinamik Psikoterapi Ders Kitabı (1. bs.). WAPP Press. 10.52982/978-3-910225-04-6.

[brb370873-bib-0007] Connor, K. M. , and J. R. T. Davidson . 2003. “Development of a New Resilience Scale: the Connor–Davidson Resilience Scale (CD‐RISC).” Depression and Anxiety 18, no. 2: 76–82. 10.1002/da.10113. 12964174

[brb370873-bib-0008] Coronado, R. A. , S. Z. George , C. J. Devin , S. T. Wegener , and K. R. Archer . 2015. “Pain Sensitivity and Pain Catastrophizing Are Associated With Persistent Pain and Disability After Lumbar Spine Surgery.” Archives of Physical Medicine and Rehabilitation 96, no. 10: 1763–1770. 10.1016/j.apmr.2015.06.003. 26101845 PMC4601931

[brb370873-bib-0009] Darlow, M. , P. Suwak , S. Sarkovich , et al. 2022. “A Pathway for the Diagnosis and Treatment of Lumbar Spinal Stenosis.” Orthopedic Clinics of North America 53, no. 4: 523–534. 10.1016/j.ocl.2022.02.002. 36208894

[brb370873-bib-0010] Ferhat, S. 2016. “Dijital dünyanın gerçekliği, gerçek dünyanın sanallığı: Bir dijital Medya Ürünü Olarak Sanal Gerçeklik.” Dijital Medya Sayısı 1: 724–746.

[brb370873-bib-0011] Firdous, T. , M. A. Siddiqui , and S. M. Siddiqui . 2019. “Frequency of Post Dural Puncture Headache in Patients Undergoing Elective Cesarean Section Under Spinal Anesthesia With Median Versus Paramedian Approach.” Anaesthesia, Pain & Intensive Care 20: 165–170.

[brb370873-bib-0014] Graffigna, G. , and S. Barello . 2018. “Patient Health Engagement (PHE) Model in Enhanced Recovery After Surgery (ERAS): Monitoring Patients' Engagement and Psychological Resilience in Minimally Invasive Thoracic Surgery.” Journal of Thoracic Disease 10, no. S4: S517. 29629198 10.21037/jtd.2017.12.84 PMC5880983

[brb370873-bib-0015] Gündoğdu Altın, N. 2022. Fibromiyalji Sendromlu Kadın Hastalarda Grup Pozitif Psikoterapi Tedavisi Ve Etkileri (Doktora tezi). İstanbul Arel Üniversitesi.

[brb370873-bib-0016] Jackson, K. L. , J. Rumley , M. Griffith , U. Agochukwu , and J. DeVine . 2020. “Correlating Psychological Comorbidities and Outcomes After Spine Surgery.” Global Spine Journal 10, no. 8: 929–939. 10.1177/2192568219889099. 32905726 PMC7485071

[brb370873-bib-0017] John, A. W. S. , J. H. Aebischer , R. Friend , and K. D. Jones . 2022. “Fibromyalgia: A Clinical Update.” Nurse Practitioner 47, no. 4: 20–30. 10.1097/01.NPR.0000820954.12040.9c. 35349514

[brb370873-bib-0018] Karaeminoğulları, O. , and U. Aydınlı . 2004. “Dejeneratif Lomber Spinal Stenoz.” TOTBİD (Türk Ortopedi ve Travmatoloji Birliği Derneği) Dergisi 3, no. 3: 1–10.

[brb370873-bib-0019] Karaokur, G. 2019. Lomber Disk Hernisi Ameliyatı Olan Hastalara Ameliyat Öncesi Verilen Eğitimin Ameliyat Öncesi Ve Sonrası Kaygı Düzeyine, Ameliyat Sonrası Ağrı Ve Memnuniyete Etkisi (Yüksek lisans tezi). Sağlık Bilimleri Enstitüsü.

[brb370873-bib-0020] Katz, J. N. , Z. E. Zimmerman , H. Mass , and M. C. Makhni . 2022. “Diagnosis and Management of Lumbar Spinal Stenosis: A Review.” JAMA 327, no. 17: 1688–1699. 10.1001/jama.2022.5162. 35503342

[brb370873-bib-0021] Kaya, F. , and H. Odacı . 2021. “Connor–Davidson Psikolojik Sağlamlık Ölçeği Kısa Formu: Türkçe'ye Uyarlama, Geçerlik Ve Güvenirlik Çalışması.” Hayef 17, no. 2: 38–54. 10.5152/HAYEF.2021.21005.

[brb370873-bib-0022] Keldal, G. 2015. “Warwick–Edinburgh Mental İyi Oluş Ölçeği'nin Türkçe Formu: Geçerlik Ve Güvenirlik Çalışması.” Journal of Happiness & Well‐Being 3, no. 1: 103–115.

[brb370873-bib-0023] Kim, H.‐J. , J.‐I. Lee , K.‐T. Kang , et al. 2015. “Influence of Pain Sensitivity on Surgical Outcomes After Lumbar Spine Surgery in Patients With Lumbar Spinal Stenosis.” Spine 40, no. 3: 193–200. 10.1097/BRS.0000000000000699. 25384051

[brb370873-bib-0024] Koraş, K. , and N. Karabulut . 2018. “The Effect of Foot Massage on Postoperative Pain and Anxiety Levels in Laparoscopic Cholecystectomy Surgery: A Randomized Controlled Experimental Study.” Journal of PeriAnesthesia Nursing 34: 551–558. 10.1016/j.jopan.2018.07.006. 30470466

[brb370873-bib-0026] Maffei, M. E. 2020. “Fibromyalgia: Recent Advances in Diagnosis, Classification, Pharmacotherapy and Alternative Remedies.” International Journal of Molecular Sciences 21, no. 21: 7877. 10.3390/ijms21217877. 33114203 PMC7660651

[brb370873-bib-0027] McGregor, A. H. , K. Probyn , S. Cro , et al. 2014. “Rehabilitation Following Surgery for Lumbar Spinal Stenosis: A Cochrane Review.” Spine 39, no. 12: 1044–1054. 10.1097/BRS.0000000000000313. 24732858

[brb370873-bib-0028] Miller, M. J. , I. Cenzer , K. E. Covinsky , E. Finlayson , P. J. Raue , and V. L. Tang . 2024. “Associations of Resilience, Perceived Control of Health, and Depression With Geriatric Outcomes After Surgery.” Annals of Surgery, Ahead of print, July 26, 10.1097/SLA.0000000000006448. PMC11762354 39056184

[brb370873-bib-0029] Müjde, S. , N. Erel , and F. Ozan . 2015. “Dejeneratif lomber dar kanal cerrahi tedavisinde Transvers Dekompresyon Yöntemi.” Acta Orthopaedica Et Traumatologica Turcica 49, no. 6: 614–619. 10.3944/AOTT.2015.14.0383. 26511687

[brb370873-bib-0030] Nasiri Takami, G. , M. Najafi , and S. Talepasand . 2020. “Comparison Group Therapy of Positive Psychotherapy and Cognitive‐Behavioral Efficacy on Adolescents' Psychological Capital With Depression Symptoms.” Positive Psychology Research 6, no. 2: 79–98. 10.22108/ppls.2021.117838.1768.

[brb370873-bib-0031] Peseschkian, H. 2023. “Positive Psychotherapy: Core Principles.” Current Psychiatry 22, no. 1: 5–9. 10.12788/cp.0317.

[brb370873-bib-0032] Petrucci, G. , G. F. Papalia , L. Ambrosio , et al. 2025. “The Influence of Psychological Factors on Postoperative Clinical Outcomes in Patients Undergoing Lumbar Spine Surgery: A Systematic Review and Meta‐Analysis.” European Spine Journal 34: 1409–1419. 10.1007/s00586-025-08733-z. 39988610

[brb370873-bib-0033] Rashid, T. , and M. P. Seligman . 2018. Positive Psychotherapy: Clinician Manual. Oxford University Press.

[brb370873-bib-0034] Seligman, M. E. , T. A. Steen , N. Park , and C. Peterson . 2005. “Positive Psychology Progress: Empirical Validation of Interventions.” American Psychologist 60, no. 5: 410–421. 10.1037/0003-066X.60.5.410. 16045394

[brb370873-bib-0035] Tennant, R. , L. Hiller , R. Fishwick , et al. 2007. “The Warwick–Edinburgh Mental Well‐Being Scale (WEMWBS): Development and UK Validation.” Health and Quality of Life Outcomes 5, no. 1: 63. 10.1186/1477-7525-5-63. 18042300 PMC2222612

[brb370873-bib-0036] Türkiye İstatistik Kurumu . 2022. “Türkiye Sağlık Araştırması 2022.” https://data.tuik.gov.tr/Bulten/Index?p=Turkiye‐Saglik‐Arastirmasi‐2022‐49747.

[brb370873-bib-0037] Wilhelm, M. , M. Reiman , A. Goode , et al. 2017. “Psychological Predictors of Outcomes With Lumbar Spinal Fusion: A Systematic Literature Review.” Physiotherapy Research International 22, no. 2: e1652. 10.1002/pri.1652. 26270324

[brb370873-bib-0038] Yekeler, B. 2022. “Lomber Dar Kanal Cerrahisinde Unilateral Yaklaşımla Bilateral Dekompresyon Yapılan Olgularda Erken Dönem Sagittal Balans Parametrelerinin Değerlendirilmesi.” PhD diss., Erciyes Üniversitesi.

[brb370873-bib-0039] Zahn, E. M. P. , D. Segelcke , and S. A. Schug . 2017. “Postoperative Pain—From Mechanisms to Treatment.” Pain Reports 2, no. 5: e588. 10.1097/PR9.0000000000000588. 29392204 PMC5770176

